# Type II alveolar epithelial cell-specific CAP1 loss is associated with spontaneous pulmonary fibrosis and altered complement 5 signaling

**DOI:** 10.1186/s12890-026-04384-y

**Published:** 2026-06-09

**Authors:** Bingbing Wang, Yunlu Gu, Jushan Zhang, Amanguli Tuerhong, Yujie Zhu, Binay Kumar Shah, Changhui Wang, Shuanshuan Xie

**Affiliations:** 1 Department of Respiratory Medicine, Shanghai Tenth People’s Hospital, Tongji University School of Medicine, No. 301, Mid Yanchang Rd., Shanghai, 200072 China; 2 Department of Respiratory Medicine, Shanghai Clinical Research and Trial Center, ShanghaiTech University, Shanghai, 201203 China; 3 Department of Respiratory Medicine, Tianyou Hospital, Tongji University School of Medicine, No.501 Zhennan Road, Putuo District, Shanghai, 200331 China

**Keywords:** CAP1, Pulmonary fibrosis, Alveolar type 2 cell, Complement 5

## Abstract

**Background:**

Cyclase-associated protein 1 (CAP1) is widely expressed in mammalian tissues; however, its physiological role in the lung remains largely unclear. This study aimed to explore the function of CAP1 in alveolar type 2 (AT2) cells and its potential association with pulmonary fibrosis.

**Methods and results:**

An inducible AT2 cell–specific CAP1 conditional knockout mouse model (CAP1^f/f^; SFTPC-Cre^ERT2^) was generated. Following tamoxifen induction, CAP1-deficient mice exhibited significantly reduced survival compared with littermate controls. A subset of knockout mice developed progressive deterioration characterized by decreased activity and weight loss, accompanied by impaired lung function, including reduced dynamic lung compliance, forced vital capacity, and forced expiratory volume at 0.05 s, while the FEV_0.05_/FVC ratio remained unchanged. Histological analysis demonstrated spontaneous fibrotic remodeling with disruption of alveolar architecture, inflammatory cell infiltration, and increased Ashcroft scores.

Transcriptomic analysis of lungs from deteriorated mice revealed enrichment of extracellular matrix–related pathways and increased expression of fibrosis-associated genes, including Col1a1, Col1a2, and Fn1. Notably, early-phase RNA sequencing performed two weeks after CAP1 deletion identified complement and coagulation cascades among the most altered pathways. Complement component 5 (C5) was significantly downregulated, which was further supported by quantitative PCR and immunohistochemistry. Reduced pulmonary C5 expression was more pronounced during the deteriorated phase. RNA sequencing of isolated CAP1-deficient AT2 cells showed decreased C5 expression together with transcriptional signatures suggestive of mitochondrial pathway alterations. CAP1 knockdown in A549 cells reduced mitochondrial membrane potential as assessed by JC-1 staining, suggesting mitochondrial-related alterations associated with CAP1 deficiency.

**Conclusions:**

These findings indicate that CAP1 deficiency in AT2 cells is associated with spontaneous fibrotic remodeling and altered complement signaling in the lung. The data support a model in which CAP1 loss may perturb AT2 cell homeostasis and local C5 expression, potentially contributing to fibrotic progression. Further studies are required to clarify the causal relationships and underlying mechanisms.

**Supplementary Information:**

The online version contains supplementary material available at 10.1186/s12890-026-04384-y.

## Introduction

Cyclase-associated protein (CAP) is a highly conserved, multidomain protein. It was first found in Saccharomyces cerevisiae more than 30 years ago [1]. The name was assigned because it was firstly recognized as an adenylyl cyclase-associated protein in yeast. In addition to the function of binding to adenylyl cyclase, CAP was also found to be involved in the activation of the RAS-family GTPase Ras-like protein 2 (RAS2) and actin cytoskeleton regulation [2–4]. There are two CAP homologs in mammals, CAP1 and CAP2. CAP1 is widely expressed in most cells and tissues, and CAP2 expression is restricted to the brain, skin, skeletal muscle, cardiac muscle and testis [5, 6].

CAP1 was found upregulated in the non-small cell lung cancer (NSCLC) tissue and higher serum CAP1 protein concentration correlated with shorter overall survival in NSCLC patients [7]. Meanwhile, CAP1 was found downregulated in COPD patients [8]. These findings indicate that CAP1 play an important role in lung.

Alveolar type 2 cells (AT2) are an important component of alveolar epithelial cells. They synthesize and secrete pulmonary surfactant, regulate the fluid balance within the alveoli, participate in immune responses, and possess stem cell characteristics, being able to proliferate and differentiate into alveolar type 1 epithelial cells [9]. Given the central position of AT2 cells in the lung tissue and the important role of CAP1 in cell functions, there are still many unknown areas regarding whether and how the knockout of CAP1 in AT2 cells affects the structural integrity of the lung tissue, gas exchange efficiency, and the process of injury repair.

This study aimed to decipher the role of CAP1 in mammalian lung. By generating alveolar type 2(AT2)-specific CAP1-conditional knockout (CKO) mice, we observed that a subset of CKO mice spontaneously developed features of pulmonary fibrosis without stimuli. Early after CAP1 deletion, transcriptomic analyses revealed alterations in the local complement system, with C5 among the most prominently downregulated genes. Further analysis of isolated CAP1-deficient AT2 cells demonstrated mitochondrial-related transcriptional changes and reduced C5 expression. Consistently, CAP1 knockdown in A549 cells resulted in reduced mitochondrial membrane potential. Taken together, our findings demonstrate that CAP1 contributes to the maintenance of AT2 cell homeostasis and complement-associated signaling, and that its loss may be associated with impaired innate immune regulation and fibrotic remodeling in the lung.

## Results

### The survival rate of CAP1 CKO mice is lowered

**Fig. 1:**
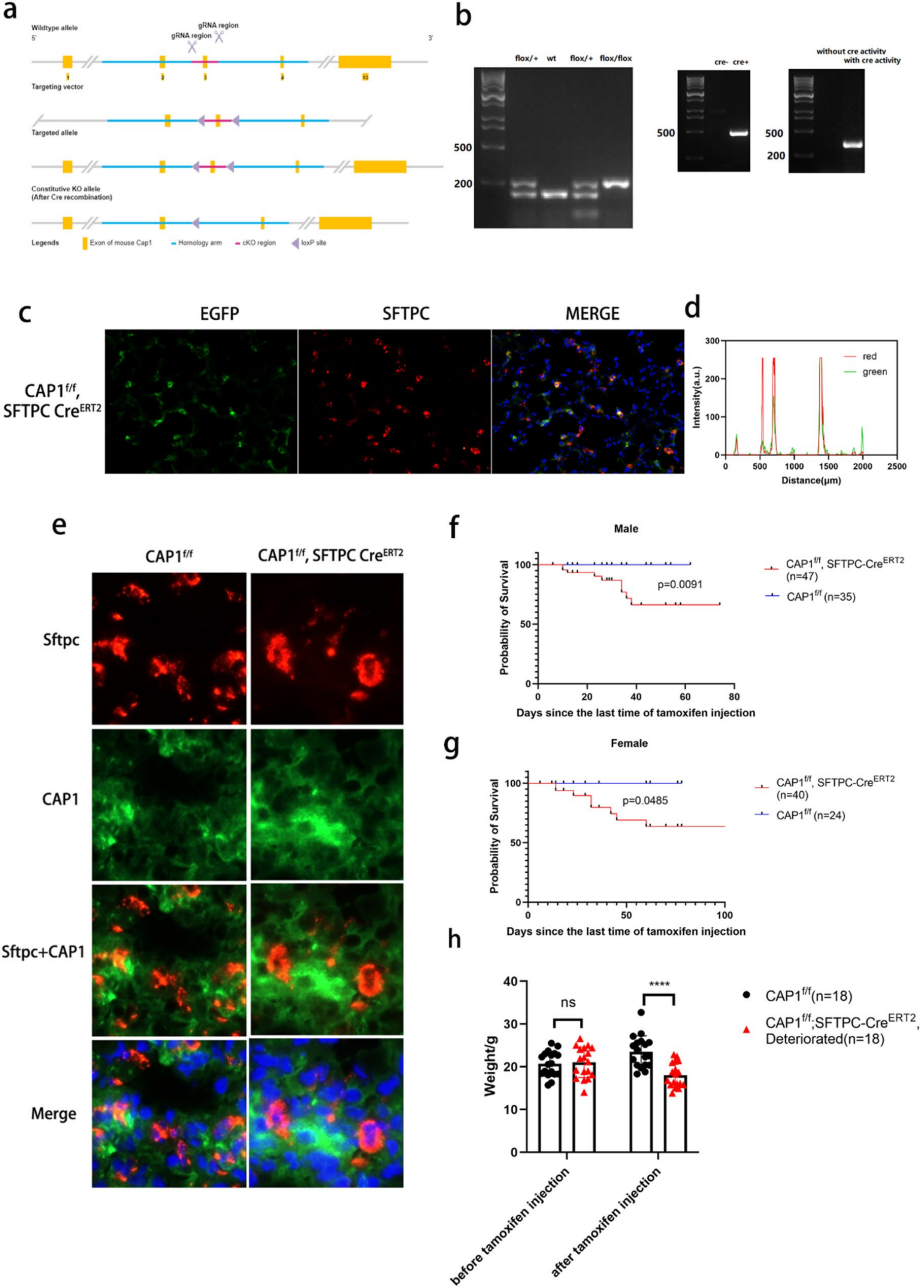
CAP1^f/f^; SFTPC-Cre^ERT2^ mice generation, validation and survival curves.** a**The strategy for creating the CAP1^f/f^; SFTPC-Cre^ERT2^ mice. **b** An example of a genotyping gel. **c** Immunofluorescence of SFTPC after administration of rAAV-CMV-DIO-EGFP-WPRE-hGH pA virus and (**d**) its co-existence analysis by Fiji (**e**) Immunofluorescence of CAP1 and SFTPC shows the absence of CAP1 in the alveolar type 2 cells in CAP1^f/f^; SFTPC-Cre^ERT2^ mice. **f** Kaplan-Meier survival curve for male mice. **g** Kaplan-Meier survival curve for female mice. Survival curves were analyzed using the log-rank (Mantel–Cox) test. p values were calculated using the log-rank test. **h **Weight of deteriorated CAP1^f/f^; SFTPC-Cre^ERT2^ mice and controls before and after tamoxifen inducible knockout. *P < 0.05; **P < 0.01; ***P < 0.001; ****P < 0.0001. A value of *P* ≥ 0.05 was considered not significant (ns)

To determine the role of CAP1 in the mouse lung, we knocked out CAP1 in AT2 in C57BL/6J mice. The strategy for creating the transgenic genotype of CAP1^f/f^ is shown in Fig. 1a. Using the CRISPR/Cas9 gene editing technology, two Loxp sequences were precisely inserted into both sides of the exon region of the CAP1 gene, and CAP1^f/+^ mice were successfully generated. The gene encoding Cre recombinase was fused with the AT2 cell-specific promoter, Surfactant Protein C (SFTPC), to construct SFTPC-Cre^ERT2^ mice. SFTPC-Cre^ERT2^ mice were crossed to CAP1^f/f^ mice to yield CAP1^f/f^; SFTPC-Cre^ERT2^ mice. PCR genotyping results of the targeted genes was shown in Fig. 1b. Tamoxifen freshly dissolved in corn oil was delivered with 4 injections at 100 mg/kg over a 1-week period to selectively delete CAP1 in AT2 cells. The rAAV - CMV - DIO - EGFP - WPRE - hGH pA was used to confirm the distribution of Cre recombinase. Figure 1c-d showed a high consistency between EGFP and SFTPC, indicating that Cre enzyme is mainly distributed in AT2 cells. Specific deletion of CAP1 in AT2 cells was confirmed by immunofluorescence (Fig. 1e).

The survival rate of male CAP1^f/f^; SFTPC-Cre^ERT2^ mice was 66.14% six weeks after inducible knockout, while that of male CAP1^f/f^ mice was 100% (*p* = 0.0091) (Fig. 1f). The survival rate of female CAP1^f/f^; SFTPC-Cre^ERT2^ mice was 63.76% two months after inducible knockout, while that of female CAP1^f/f^ mice was 100% (*p* = 0.0485) (Fig. 1g). Mice in the moribund stage showed symptoms of reduced activity and weight loss. There was no significant difference in the survival rates between female and male CKO mice.

The body weight records captured the weights of 18 CAP1^f/f;^ SFTPC-Cre^ERT2^ mice before death. Prior to tamoxifen injection, there was no significant difference in body weight between the CAP1^f/f^; SFTPC-Cre^ERT2^ mice and the control CAP1^f/f^ mice (21.05 ± 3.48 g vs. 20.68 ± 2.85 g, *p* > 0.05). However, following tamoxifen-induced knockout, the body weight of CAP1^f/f^; SFTPC-Cre^ERT2^ mice declined significantly compared to the control group shortly before death (18.07 ± 2.74 g vs. 23.53 ± 3.61 g, *p* < 0.0001) (Fig. 1h).

### AT2-specific CAP1 deficiency is associated with spontaneous fibrotic remodeling and reduced survival

We categorized CAP1^f/f^; SFTPC-Cre^ERT2^ mice with decreased activity and significant weight loss as CKO deteriorated group, and CAP1^f/f^ mice from the same cage as the control group. Pulmonary function was measured in the two groups. Several key respiratory function parameters showed significant difference between the groups (Table 1). CKO deteriorated mice had 58% less dynamic lung compliance (*p* = 5.9*10^− 5^) than control group. FVC in CKO deteriorated mice was significantly lower than that in control mice (0.50 vs. 0.69 mL, *p* = 0.001). FEV_0.05_ in CKO deteriorated mice was also significantly lower than that in control mice (0.14 vs. 0.17 mL, *p* = 0.037). While FEV_0.05_/FVC showed no significant difference between CKO deteriorated mice and controls.

**Table 1:** Pulmonary function parameters of CAP1 CKO Deteriorated mice and controls

pulmonary function parameter	unit	CAP1^f/f^; SFTPC-Cre^ERT2^, Deteriorated		CAP1^f/f^			*p* value
		mean	sd	mean	sd		
RI	cmH_2_O·s/mL	3.61	0.71	1.10	0.11	1.88691E-06	
Cdyn	mL/cmH_2_O	0.012	0.005	0.028	0.005	5.86825E-05	
PIF	mL/s	1.22	0.49	2.15	0.24	0.001304554	
PEF	mL/s	1.86	0.72	3.12	0.45	0.003600094	
VC	mL	0.14	0.06	0.28	0.03	0.000298939	
MVV	mL	11.99	4.16	24.81	2.97	0.000120847	
Penh		0.87	0.38	1.29	0.21	0.038529281	
FVC	mL	0.50	0.07	0.69	0.08	0.001046197	
FEV_0.05_	mL	0.14	0.02	0.17	0.02	0.037012252	
FEV_0.1_	mL	0.45	0.05	0.56	0.06	0.005560615	
FEV_0.1_/FVC%	%	88.97	4.11	81.11	5.39	0.01487707	
FEF25%	mL/s	5.52	0.68	7.06	0.82	0.004273829	
FEF50%	mL/s	6.94	0.87	8.56	1.05	0.012760001	
FEF25_75%	mL/s	6.58	0.82	8.04	0.99	0.016496365	
FEF25_75/FVC%	%	1312.74	27.94	1170.28	78.19	0.001226358	
FMFT	s	0.038	0.001	0.043	0.003	0.002117262	
MMF	ml/s	6.58	0.82	8.05	0.99	0.01603165	
F50/F25%	%	1.26	0.01	1.21	0.03	0.003675578	
PEF	mL/s	6.96	0.87	8.57	1.05	0.013323798	
FET	s	0.12	0.01	0.14	0.01	0.005420978	

*Abbreviations*: *RI* Inspiratory resistance, *Cdyn* Dynamic lung compliance, *PIF* Peak Inspiratory Flow, *PEF* Peak Expiratory Flow, *VC* Vital Capacity, *MVV* Maximum Voluntary Ventilation, *Penh* Enhanced Pause, *FVC* Forced Vital Capacity, *FEV*_*0.05*_ Forced Expiratory Volume at 0.05 s, *FEV*_*0.1*_ Forced Expiratory Volume at 0.1 s, *FEF25%* Forced Expiratory Flow at 25% of FVC, *FEF50%* Forced Expiratory Flow at 50% of FVC, *FEF25_75%* Forced Expiratory Flow between 25% and 75% of FVC, *FMFT* Forced Mid-Flow Test, *MMF* Maximum Mid-Flow, *F50/F25%* Ratio of Forced Expiratory Flow at 50% to 25% of FVC, *PEF* Peak Expiratory Flow, *FET* Forced Expiratory Time

**Fig. 2:**
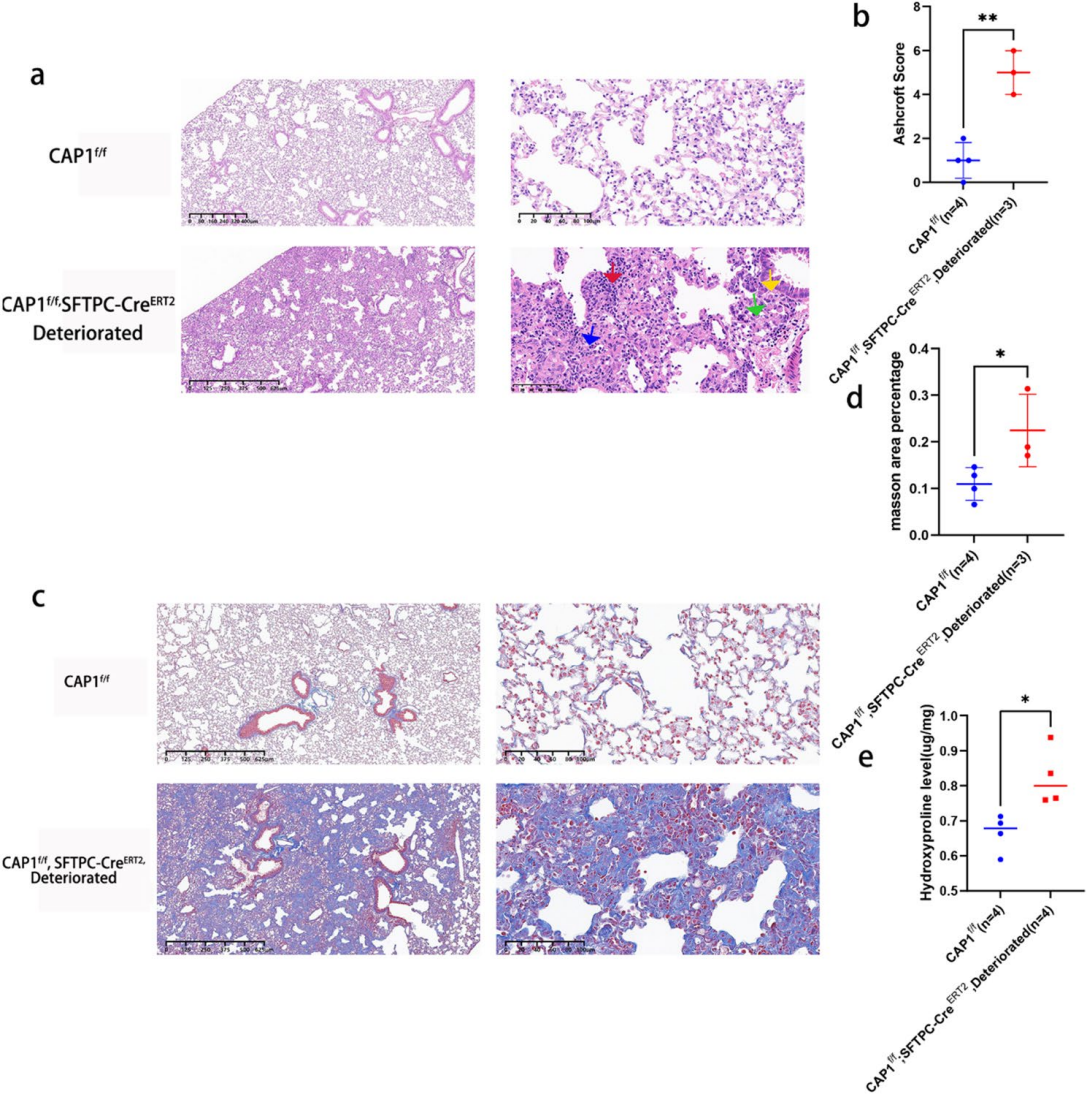
Lung histology in CAP1-CKO deteriorated mice.** a** HE staining of deteriorated lungs shows alveolar wall hyperplasia (blue arrow), reduced alveolar space, diffuse inflammatory cell infiltration (red arrow), and mild bronchial wall thickening (yellow arrow). **b** Ashcroft score of the lung pathology between CAP1-CKO, deteriorated mice and control mice. **c** Masson staining of lungs. **d** Quantitative analysis of the Masson staining findings. **e **Hydroxyproline level of between CAP1-CKO, deteriorated mice and control mice.*P < 0.05; **P < 0.01; ***P < 0.001; ****P < 0.0001. A value of *P* ≥ 0.05 was considered not significant (ns)

Then we histologically analyzed the lungs from the two groups. Compared to the normal morphology and structure of the lung tissue with only a small amount of inflammatory cell infiltration in control mice, fibrosis was observed in the CKO deteriorated mice lung tissue accompanied with significant destruction of the lung structure, along with diffuse infiltration of inflammatory cells and the significantly thickened bronchial wall (Fig. 2a). The lung histological Ashcroft score of the CKO deteriorated mice was significantly higher than that of the control mice (5.0 ± 1.0 vs. 1.0 ± 1.0, *p*<0.01) (Fig. 2b). Based on the lung histology analysis, collagen fibers were evaluated by Masson staining (Fig. 2c). Quantity analysis showed that Masson area percentage of the CKO deteriorated mice was significantly higher than that of the control mice (0.22 ± 0.06 vs. 0.11 ± 0.03, *p*<0.05) (Fig. 2d). Hydroxyproline level of the CKO deteriorated mice was significantly higher than that of the control mice༈0.82 ± 0.07 vs. 0.66 ± 0.05, *p*<0.05 (Fig. 2e).

**Fig. 3:**
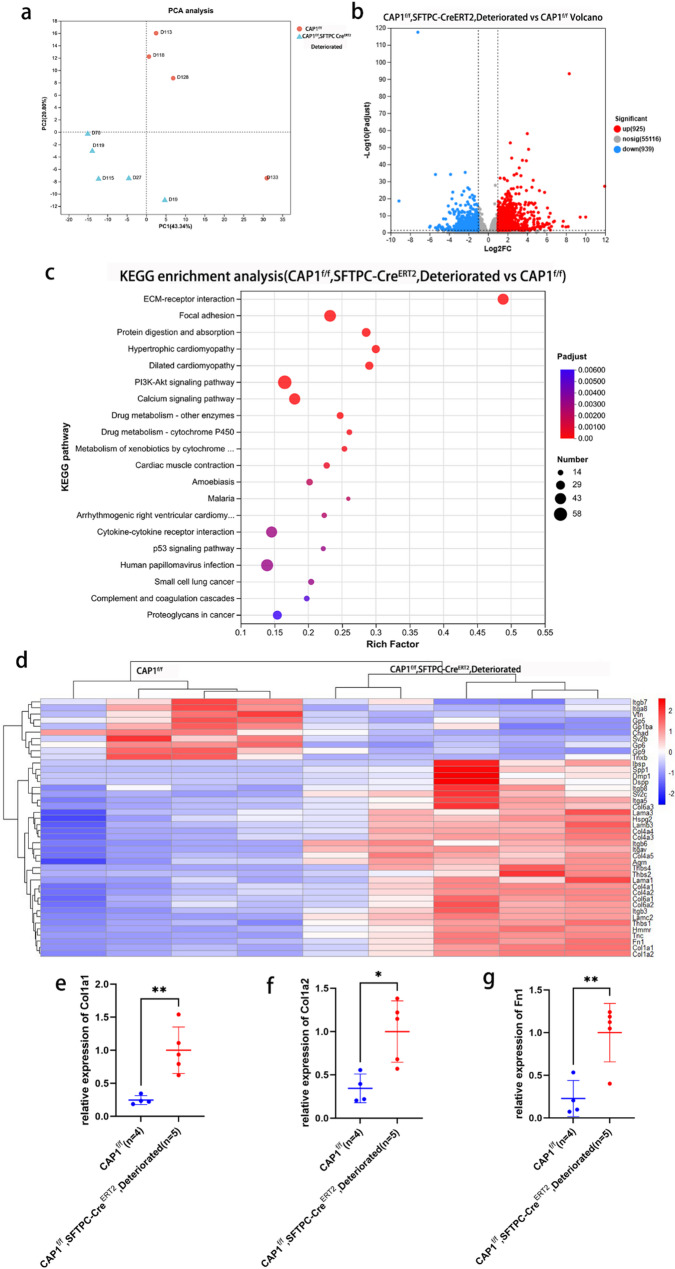
Alteration of genes and pathways in the lung of CAP1-CKO deteriorated mice. RNA was isolated from the lung of CAP1^f/f^; SFTPC-Cre^ERT2^, deteriorated mice (*n* = 5) and age-matched CAP1^f/f^ mice (*n* = 4). **a** PCA analysis. **b** Volcano plots showing differentially expressed genes in CAP1^f/f^;SFTPC-CreERT2 deteriorated lungs compared with control. Red dots indicate significantly upregulated genes in CAP1-CKO deteriorated lungs; blue dots indicate downregulated genes (*P* < 0.05). **c **KEGG enrichment analysis of CKO deteriorated group and control. **d** Heatmap of significantly regulated genes in the ECM-receptor interaction pathway in the lungs of CKO deteriorated mice compared with control mice (**e-g**) qPCR analysis of representative genes of ECM-receptor interaction pathway. Bars represent mean values ± SEM. P value between groups was based on Student’s t test. *P < 0.05; **P < 0.01; ***P < 0.001; ****P < 0.0001. A value of *P* ≥ 0.05 was considered not significant (ns)

To identify differences in gene expression between CKO deteriorated mice and control mice, we carried out RNA sequencing (RNA-seq) analysis in isolated lungs. Principal Component Analysis plot clearly demonstrated that the two groups were distinctly separated (Fig. 3a). RNA-seq identified 925 upregulated genes and 939 downregulated genes in CKO deteriorated mice (Fig. 3b). According to the KEGG enrichment pathway analysis (Fig. 3c), the top significantly influenced pathways were ECM-receptor interaction. Most genes involved in ECM-receptor interaction pathway including Col1a1, Col1a2, Fn1, lox were upregulated (Fig. 3d), which is coincident with the pathological change of the pulmonary interstitium. qPCR also confirmed the upregulation of Col1a1, Col1a2 and Fn1 (Fig. 3e-g).

### Reduced local C5 expression precedes fibrotic remodeling in CAP1-deficient lungs

**Fig. 4:**
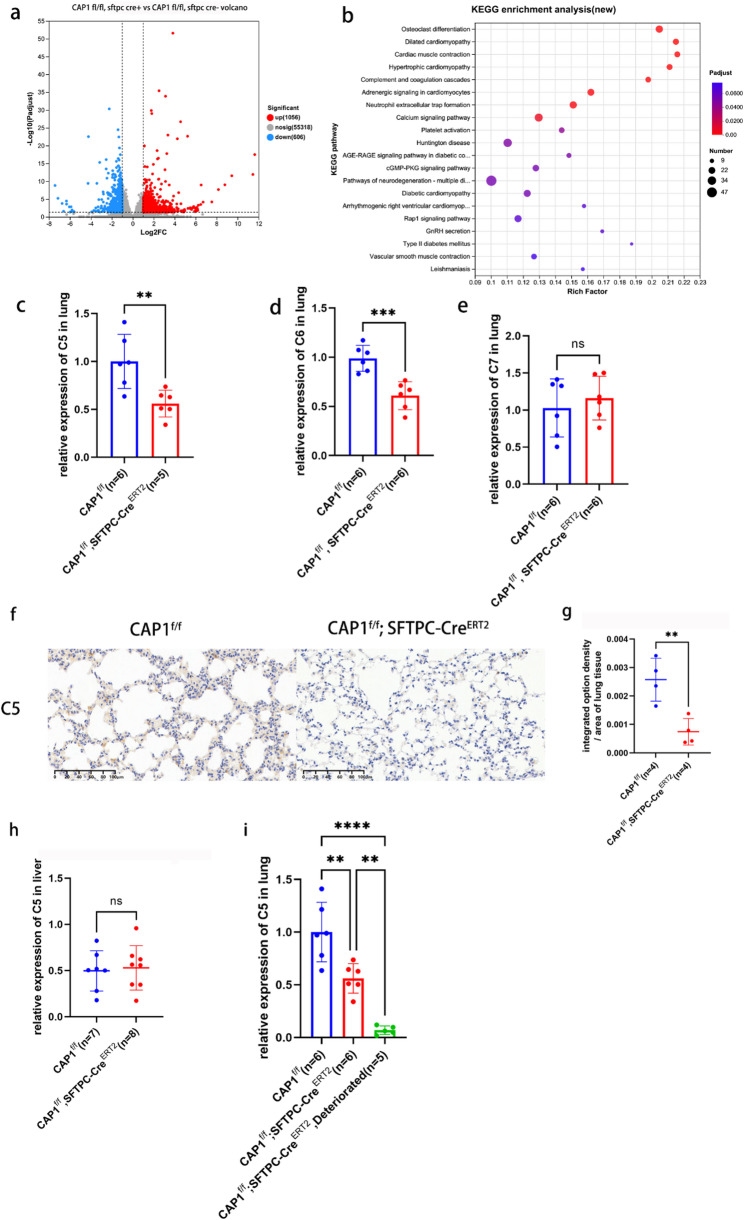
Genes and pathways alterations in the lung of CAP1-CKO mice 2 weeks after inducible knockout.** a** Heatmap and (**b**) KEGG enrichment analysis of CAP1^f/f^; SFTPC-Cre^ERT2^ mice compared with control 2 weeks after inducible knockout. Red dots indicate significantly upregulated genes in CAP1-CKO lungs; blue dots indicate downregulated genes (*P* < 0.05). C5 (**c**), C6 (**d**), C7 (**e**) expression in the lung of CKO mice was validated by qPCR. **f**,** g **Distribution and quantification of C5 protein in the lung were evaluated by IHC. **h** Relative expression of C5 in the liver was investigated by qPCR. **i**, C5 expression in lung of control, CAP1^f/f^; SFTPC-Cre^ERT2^ at 2 weeks after inducible knockout and deteriorated phase was explored by qPCR. *P < 0.05; **P < 0.01; ***P < 0.001; ****P < 0.0001. A value of *P* ≥ 0.05 was considered not significant (ns)

To explore gene expression differences in the lung at an early time point prior to overt fibrotic remodeling, we harvested the lung of CAP1^f/f^; SFTPC-Cre^ERT2^ mice and CAP1^f/f^ mice 2 weeks after the final tamoxifen injection for RNA-seq analysis, obtaining 1056 upregulated genes and 606 downregulated genes in CKO mice (Fig. 4a). Among the top 20 significant pathways analyzed by KEGG, complement and coagulation cascades were found to be most significant pathway related to respiratory (Fig. 4b). Table 2 shows the expression level of differentially expressed genes of the complement and coagulation cascades pathway. Among them, Complement 5(C5), was the most significant differently expressed gene. C5 is a component of the complement system and a part of the innate immune system, plays an important role in inflammation, host homeostasis, and host defense against pathogens.

**Table 2:** Summary of significantly regulated genes in the complement and coagulation cascades pathway

	Gene	Function	CAP1^f/f^ TPM	CAP1^f/f^; SFTPC-Cre^ERT2^ TPM	log2FC (CKO vs. Control)	P value
Downregulated	C5	Complement 5	234.0866667	85.59333333	-1.921589238	3.78382E-15
	Thbd	Thrombomodulin	483.67	196.8866667	-1.034288039	1.99786E-09
	Serpind1	serine (or cysteine) peptidase inhibitor, clade D, member 1	2.536666667	0.426666667	-2.318269289	9.04931E-09
	C7	complement component 7	120.7233333	44.85333333	-1.187008612	4.46401E-06
	Itgam	integrin alpha M	16.57666667	5.53	-1.319914114	1.32405E-05
	C6	complement component 6	30.06	11.44333333	-1.044877436	0.000174062
	F5	coagulation factor V	1.693333333	0.696666667	-1.022111722	0.001152828
	Itgb2	integrin beta 2	124.2466667	25.67333333	-2.035434734	0.001924592
	F10	coagulation factor X	11.74	3.54	-1.441600079	0.001948444
	C3ar1	complement component 3a receptor 1	3.946666667	1.306666667	-1.290549066	0.005229218
	F7	coagulation factor VII	19.80333333	6.256666667	-1.376857526	0.006668675
Upregulated	F3	coagulation factor III	44.86	84.65333333	1.149137765	4.62058E-07
	Kng2	kininogen 2	0.393333333	2.7	2.880585564	8.37077E-07
	Fgg	fibrinogen gamma chain	1.906666667	5.856666667	1.85633045	0.000115733
	Bdkrb1	bradykinin receptor, beta 1	2.973333333	5.36	1.162209318	0.001156125
	Fga	fibrinogen alpha chain	0.4	1.22	1.808792849	0.001374965
	Proc	protein C	0.476666667	1.386666667	1.885444602	0.010675166

log2FC values represent CAP1^f/f^; SFTPC-Cre^ERT2^ (CKO) relative to CAP1^f/f^ (Control). Positive values indicate higher expression in CKO group. Downregulated: lower expression in CKO relative to Control; Upregulated: higher expression in CKO relative to Control

To confirm the expression level of the genes identified by RNA-seq, we used qPCR to quantify the expression of C5, C6, C7. qPCR revealed that C5 and C6 mRNA were significantly lower in the lung of CAP1^f/f^; SFTPC-Cre^ERT2^ group while C7 mRNA was not significantly different between groups (Fig. 4c-e). IHC analysis of C5 protein expression in lung tissue showed that the integrated optical density (IOD) measured by ImageJ software was significantly lower in CAP1^f/f^; SFTPC-Cre^ERT2^ mice compared to CAP1^f/f^ mice (0.0007 ± 0.0004 vs. 0.0026 ± 0.0007, *p* < 0.01) (Fig. 4f and g). Knowing that C5 is produced primarily by the liver and released into the bloodstream as part of the immune system, we detected the expression level of C5 in the liver. However, the mRNA of C5 in the liver were similar between the CAP1^f/f^; SFTPC-Cre^ERT2^ group and CAP1^f/f^ group (Fig. 4h). Further analysis enrolled C5 mRNA change in the lung of CKO deteriorated group, which showed that compared with CAP1^f/f^ mice, the lung C5 mRNA levels in CAP1^f/f^; SFTPC-Cre^ERT2^ mice were significantly reduced at the early stage after knockout (0.56 ± 0.13 vs. 1.00 ± 0.26, *p* < 0.01), and the reduction was even more pronounced at the deteriorated phase of disease progression (0.07 ± 0.04 vs. 1.00 ± 0.26, *p* < 0.0001) (Fig. 4i).

### AT2-specific CAP1 deficiency is associated with reduced C5 expression

**Fig. 5:**
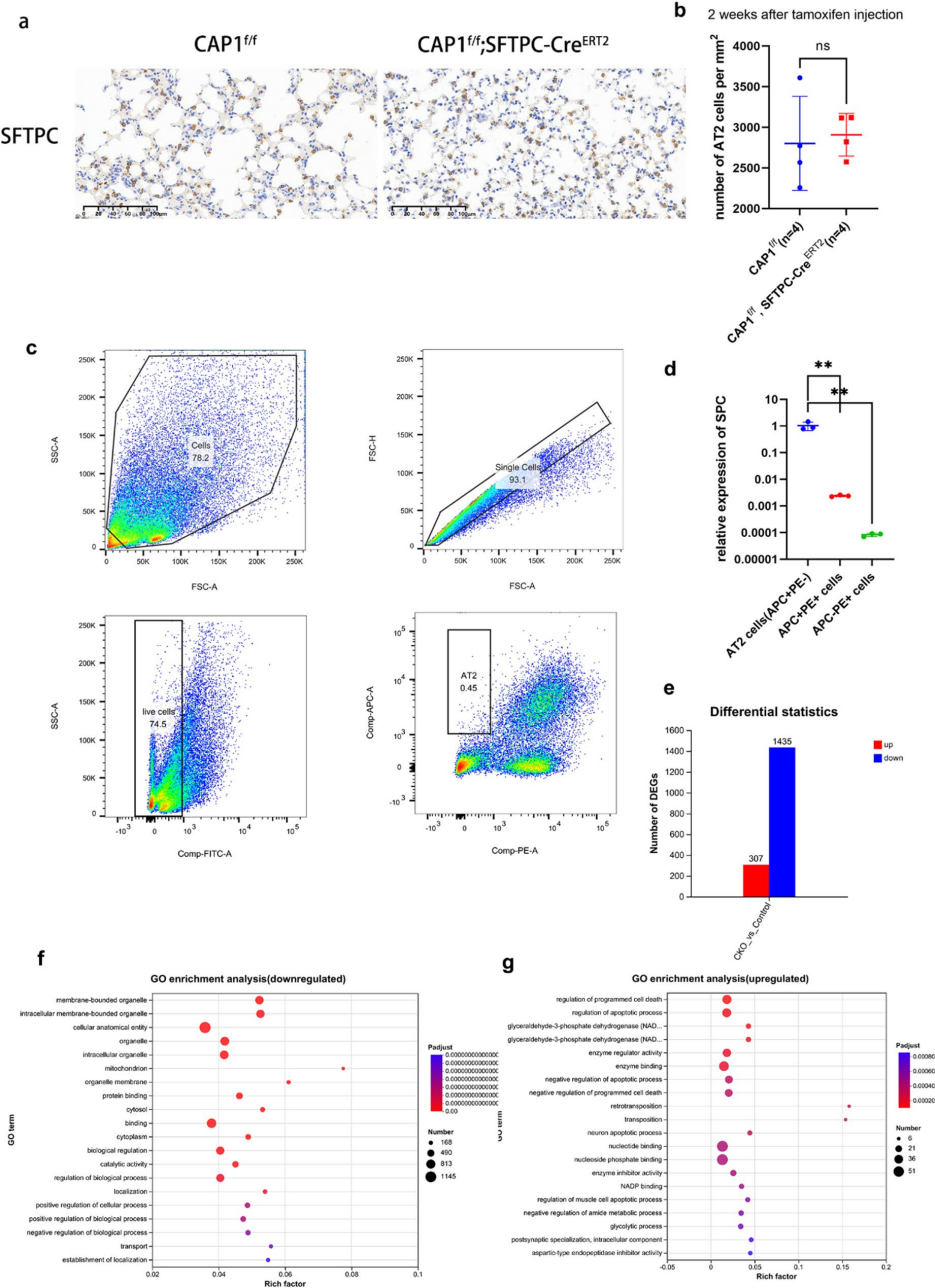
The sorting strategy for isolating AT2 cells and RNA expression changes in isolated AT2 cells. **a**,** b **The number of AT2 cells 2 weeks after inducible knockout was evaluated and calculated by IHC of SFTPC staining. Lungs from CAP1^f/f^; SFTPC-Cre^ERT2^ mice were digested into a single-cell suspension. **c** Alive EpCAM positive cells (APC) with negative CD31, CD45, podoplanin, Sca1 and CD24 (all PE) were regarded as AT2 cells. **d** qPCR revealed the SPC expression of sorted APC + PE- cells, APC + PE+ cells and APC-PE+ cells. **e** Statistical chart showing differences in alteration of expression level in AT2 cells of CAP1-CKO mice 2 weeks after inducible knockout. GO enrichment analysis of downregulated (**f**) and upregulated (**g**) genes in AT2 cells of CAP1-CKO mice 2 weeks after inducible knockout compared with control group. *P < 0.05; **P < 0.01; ***P < 0.001; ****P < 0.0001. A value of *P* ≥ 0.05 was considered not significant (ns)

Considering that systemic knockout of CAP1 in mice is lethal, we speculated that the deletion of CAP1 in AT2 cells might be associated with cell death and a subsequent reduction in their numbers, thereby contributing to pulmonary fibrosis. Therefore, we examined whether the number of AT2 cells in the lungs changed 2 weeks after tamoxifen injection in CAP1^f/f^; SFTPC-Cre^ERT2^ mice. We performed IHC staining for SFTPC in lung tissue and quantified the number of AT2 cells per unit area (Fig. 5a). The results showed no significant difference in AT2 cell numbers between CAP1^f/f^ mice and CAP1^f/f^; SFTPC-Cre^ERT2^ mice (2801.84 ± 500.87/mm² vs. 2908.06 ± 227.12/mm², *p* > 0.05) (Fig. 5b). We therefore isolated AT2 cells from the mice. The gating strategy is shown in Fig. 5c. qPCR also confirmed the isolated AT2 cells (Fig. 5d). TPM of Hc/C5 in AT2 cells was 0 in CKO group and 68.03 in CAP1^f/f^; SFTPC-Cre^ERT2^ group(*p*<0.01) (Supplemental Table 2). RNA-seq identified 1435 downregulated genes and 307 upregulated genes in AT2 cells from the CAP1^f/f^; SFTPC-Cre^ERT2^ mice 2 weeks after tamoxifen injection (Fig. 5e). GO enrichment analysis revealed that the membrane-bounded organelles, specifically mitochondria, were downregulated, and the regulation of programmed cell death was upregulated (Fig. 5f and 5g).

**Fig. 6:**
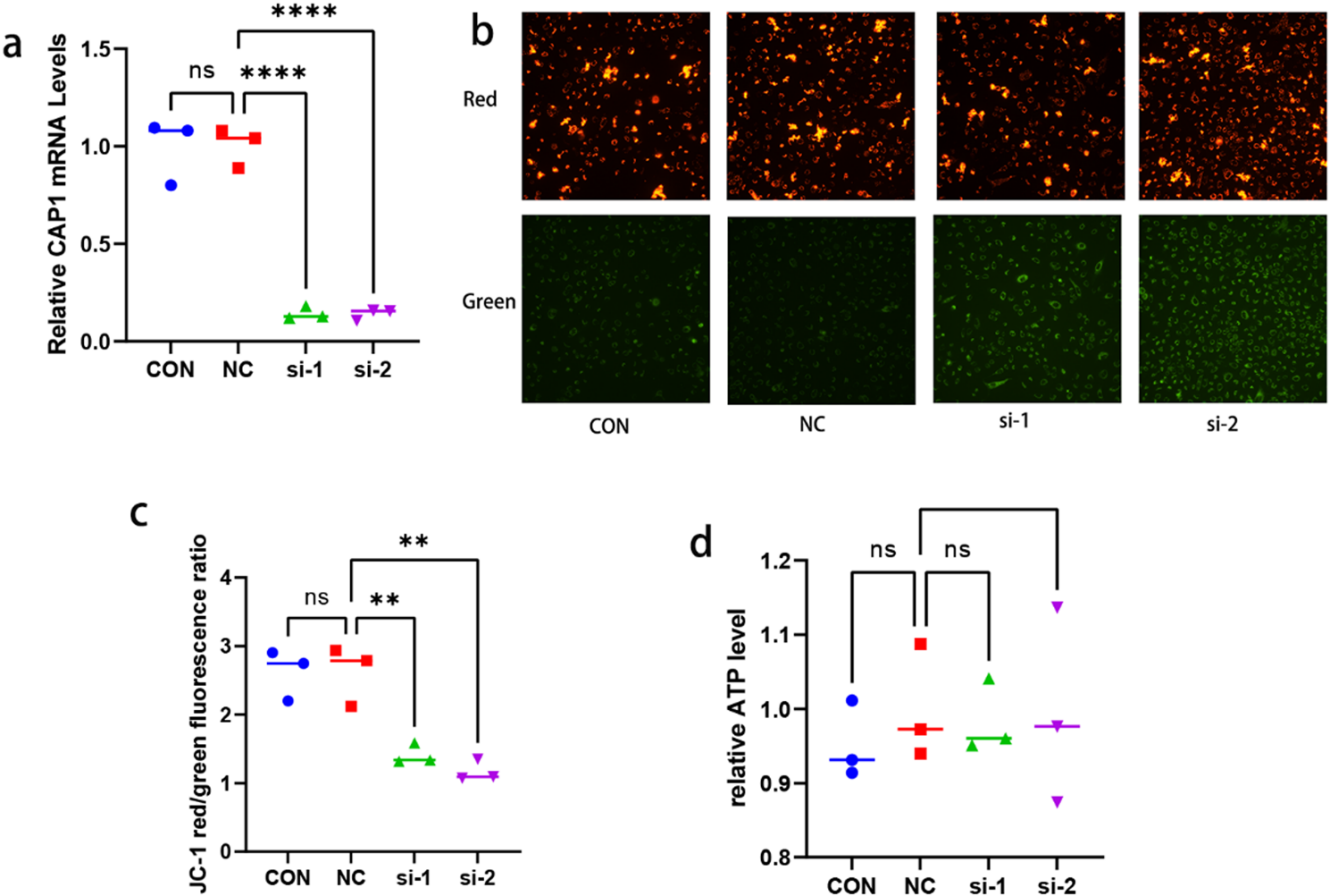
CAP1 knockdown is associated with mitochondrial-related alterations in A549 cells. **a** qPCR validation of CAP1 knockdown efficiency in A549 cells following siRNA transfection. **b** Representative JC-1 staining images in control, negative control siRNA(NC) and siCAP1-treated A549 cells. Red fluorescence indicates JC-1 aggregates, and green fluorescence indicates JC-1 monomers. **c** Quantification of JC-1 red/green fluorescence ratio in A549 cells. CAP1 knockdown significantly reduced mitochondrial membrane potential compared with the NC group. **d** Intracellular ATP levels normalized to total protein in control, NC and siCAP1-treated A549 cells. No significant difference was observed between groups under the current experimental conditions. *P < 0.05; **P < 0.01; ***P < 0.001; ****P < 0.0001. A value of *P* ≥ 0.05 was considered not significant (ns).

To further investigate whether CAP1 deficiency is associated with mitochondrial-related alterations in alveolar epithelial cells, CAP1 knockdown was performed in A549 cells using siRNA. qPCR analysis confirmed efficient knockdown of CAP1 expression, with more than 80% reduction compared with the negative control(NC) group. Mitochondrial membrane potential was subsequently assessed using JC-1 staining. Compared with the NC group, CAP1 knockdown cells exhibited a significantly reduced JC-1 red/green fluorescence ratio(*p*<0.01), indicating altered mitochondrial membrane potential following CAP1 deficiency. In addition, intracellular ATP levels normalized to total protein were measured. However, no significant difference in ATP levels was observed between the siCAP1 groups and the NC group under the current experimental conditions (Fig. 6).

## Discussion

**Fig. 7:**
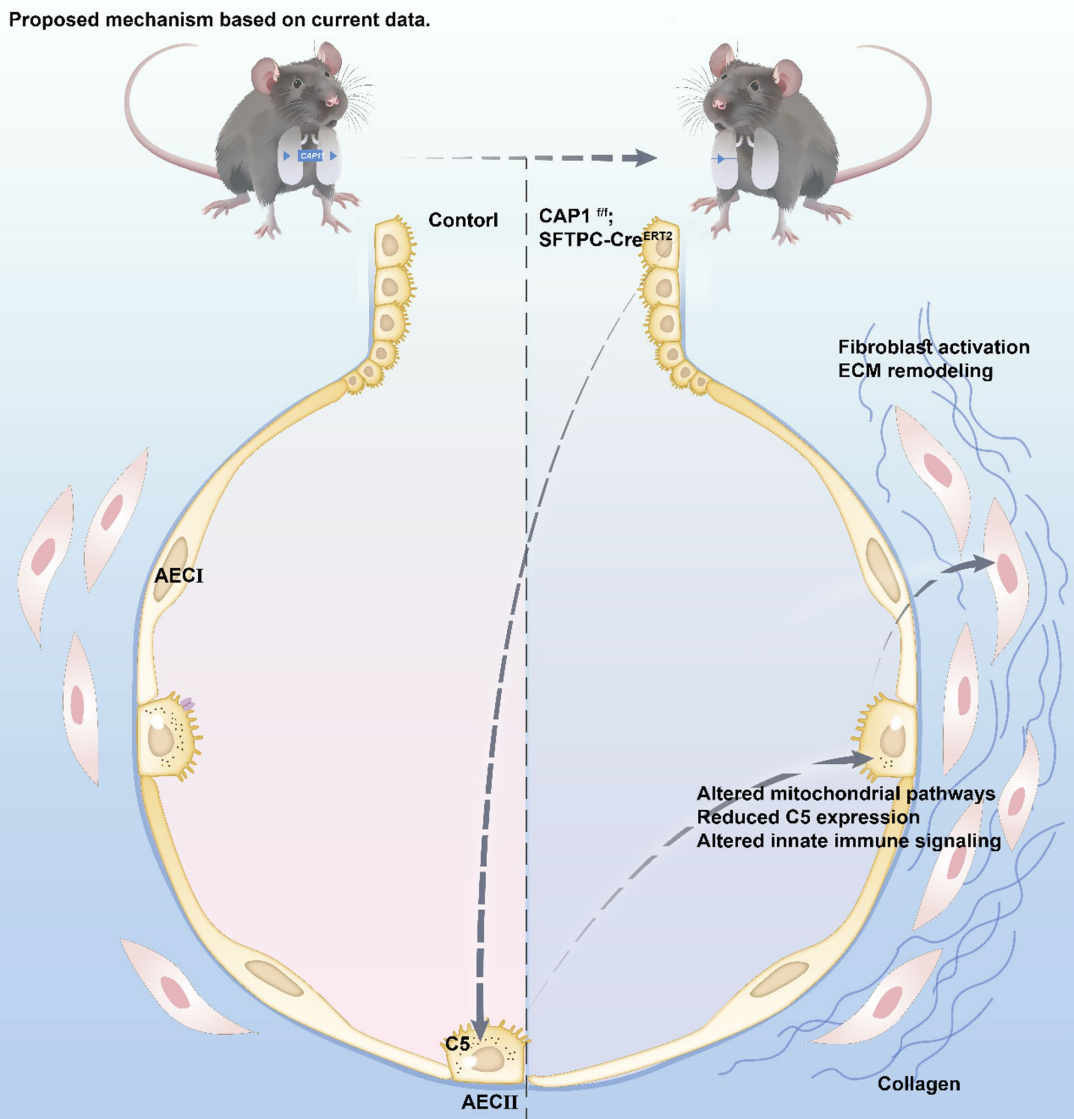
Proposed model illustrating the association between AT2-specific CAP1 deficiency and fibrotic remodeling

Knowing that AT2 plays a crucial role in maintaining alveolar function and homeostasis, we generated AT2-specific CAP1 CKO mice to investigate the physiological role of CAP1 in the lung. CAP1 deletion in AT2 cells was associated with reduced survival in both male and female mice, with mortality occurring between 2 and 9 weeks after tamoxifen induction. Functional assessment, Masson staining, and bulk RNA-seq analysis of lungs from deteriorated CKO mice demonstrated features consistent with fibrotic remodeling. Importantly, early-phase lung RNA-seq performed prior to overt fibrotic changes revealed significant downregulation of complement and coagulation pathways, including reduced C5 expression compared with controls. Consistently, isolated AT2 cells from early-phase CKO mice also exhibited decreased C5 expression following CAP1 deletion. In addition, transcriptomic analysis suggested downregulation of mitochondrial-related pathways in CAP1-deficient AT2 cells. Supporting these findings, CAP1 knockdown in A549 cells resulted in reduced mitochondrial membrane potential as assessed by JC-1 staining (Fig. 7).

The phenotypic features observed in CAP1-CKO mice—including collagen deposition, reduced lung compliance (RI, Cdyn, FVC), and progressive mortality—resemble key pathological characteristics of idiopathic pulmonary fibrosis (IPF), a disease associated with poor prognosis and a median survival of 3–5 years after diagnosis [10]. As its name alludes to, the etiology of IPF is largely unknown. Typically genetic predisposition is supposed to be associated with mutations in surfactant proteins, telomere network and immune-modulators and inflammation [11–15]. Mutation in immune-modulators and inflammation are likely to lead to dysregulation of the immune response and chronic inflammation. Although the CAP1-CKO model shares certain features with immune-related mechanisms implicated in IPF, there is currently no consistent evidence demonstrating altered CAP1 expression in IPF lung tissue or specifically in AT2 cells. This suggests that CAP1-related mechanisms identified in our study may reflect context-dependent regulation of AT2 cell homeostasis rather than a direct recapitulation of known human genetic alterations in IPF.

CAP is a key regulator of actin dynamics, which is essential for various cellular processes including cytoskeletal organization and cell motility. Several CAP1 targeted mouse models have been investigated. Embryonic lethality was observed in systemic CAP1 KO mice, while systemic heterozygous CAP1-KO mice enabled Jang et al.l to identify CAP1 as a new binding partner of Proprotein convertase subtilisin/kexin type-9(PCSK9) and a key mediator of caveolae-dependent endocytosis and lysosomal degradation of LDLR [16]. Brain-specific CAP1-KO impaired neuronal connectivity and caused perinatal lethality, with CAP1 acting as a novel actin regulator in growth cones critical for neuron connectivity [17]. In our research, we injected tamoxifen to induce CAP1 knockout from AT2 when mice grew to 6–8 weeks to ensure lung maturation. A week after tamoxifen injection, CAP1 was deleted from AT2 cells, which was confirmed by immunofluorescence of the lung tissue and smart-seq of isolated AT2 cells. Two weeks after tamoxifen injection, transcriptomic analysis revealed downregulation of mitochondrial-related pathways and enrichment of apoptotic process–associated genes in AT2 cells. CAP1 knockdown in A549 cells resulted in reduced mitochondrial membrane potential. These findings suggest potential alterations in mitochondrial homeostasis and cell survival pathways following CAP1 deletion. Our data therefore support a role for CAP1 in maintaining AT2 cellular homeostasis, consistent with previous findings by Yang et al., who reported that the resistin–CAP1 axis regulates mitochondrial function in animal models of obesity [18].

At the same time, we figured out that the most obvious change in the early knockout phase in CKO lung was complement and coagulation cascades, among which C5 was the most obvious downregulated factor. Previous studies showed that AT2 cells protected the lung against infection by secreting antimicrobial peptides and proteins in the innate immunity [19, 20]. Early in 1988, Strunk et al. found that C5 was synthesized locally in the lung in alveolar type II epithelial cells [21]. Recently, Chaudhary et al. explored the expression of complement genes through computational analysis of single cell RNA sequencing data from the lung, and found that C5 was highly expressed in ~ 75% of AT2 cells [22]. These observations, together with our RNA-seq data from isolated CAP1-deficient AT2 cells, support the notion that CAP1 deletion is associated with reduced C5 expression in AT2 cells. The complement is a part of the innate immune system, playing a crucial role in defending pathogens. C5 signaling, through the actions of C5a and C5b, plays a critical role in the immune response by promoting inflammation, cell recruitment, pathogen lysis, and modulation of immune cell function. Reduced local C5 expression may have implications for AT2-mediated immune homeostasis. In the deteriorated phase of AT2-specific CAP1-CKO mice, we observed collagen accumulation and impaired pulmonary function, consistent with progressive fibrotic remodeling.

This study has several limitations. Firstly, the cell sorting strategy used in this study was adapted from the method described by A. J. Paris et al. (2020) [23], which has been widely applied in related studies. However, our gating strategy validation only demonstrated an enrichment of AT2 cells, without providing information on the presence or proportion of potential contaminating cell populations. Second, the observed mitochondrial-related transcriptional alterations and reduced C5 expression in CAP1-deficient AT2 cells are correlative and do not establish causality. Interestingly, although CAP1 knockdown significantly altered mitochondrial membrane potential as assessed by JC-1 staining, intracellular ATP levels did not show a significant change under the current experimental conditions. This discrepancy may suggest that mitochondrial membrane potential alterations occur prior to overt impairment of ATP production.

## Materials and methods

### Ethical approval of the study protocol

All animal experiments were conducted in accordance with the ethical guidelines and regulations for the care and use of laboratory animals. Animals were housed in a specific pathogen-free (SPF) facility under controlled conditions of temperature (22 ± 2 °C), humidity (50 ± 10%), and a 12-hour light/dark cycle.

### Generation of CAP1 conditional knockout mice

CAP1 conditional genetically targeted (CAP1^f/+^) mice and SFTPC-Cre^ERT2^ mice were commercially generated by Cyagen Biosciences (Suzhou, China). The Loxp site was knocked into the genomic position to get embryonic stem cells to obtain CAP1^f/f^ mice. Thereafter, the cyclization recombination enzyme (Cre) was specifically expressed in AT2 cells using the promoter and enhancer of Sftpc [24]. Then, SFTPC-Cre^ERT2^ mice were crossed to CAP1^f/f^ mice to yield CAP1^f/f^; SFTPC -Cre^ERT2^ mice. After tamoxifen(catalog number, T5648; SigmaMillipore, Burlington, MA, USA) was freshly dissolved in corn oil and then delivered with 4 injections at 100 mg/kg over a 1-week period at the age of 6–8 weeks, and the sequence between the two Loxp sites was sheared off, allowing deletion of the CAP1 fragment.

### Genotyping

To confirm the presence of the genetic modification, genomic DNA was extracted from the mouse tail biopsies using the standard protocol of One Step Mouse Genotyping Kit(Vazyme Biotech Co., Ltd., Nanjing, China). Polymerase chain reaction (PCR) was performed to amplify specific regions of the genome using primers designed to target the inserted transgene. Specific primers are shown in the supplemental material.

### Administration of AAV virus

The mouse was deeply anesthetized by intraperitoneal injection of 5% pentobarbital sodium (50 mg/kg). Place the anesthetized mouse in a supine position and gently fix its head to ensure that the nostrils are facing upward. Slowly instill the rAAV-CMV-DIO-EGFP-WPRE-hGH pA (serotype 6) viral particles(2.5 × 10^12^vg/ml, 20uL each mouse) into both nostrils in several portions, 2–5 µL each time, ensuring that the liquid is inhaled by the mouse.

### Pulmonary function

Spirometry was performed using an AniRes2005 Lung Function System (version 3.5; Bestlab Technology Co., Ltd., Chengdu, China) according to the manufacturer’s instructions. The mouse was deeply anesthetized by intraperitoneal injection of pentobarbital sodium (50 mg/kg), tracheotomized, and connected to a computer-controlled ventilator via the tracheal cannula. Respiratory function parameters including inspiratory resistance (RI), expiratory resistance (RE), dynamic compliance (Cdyn), forced vital capacity (FVC), forced expiratory volume in 0.05 s (FEV_0.05_), and FEV_0.05_/FVC were collected.

### Lung tissue harvest

Prior to lung tissue harvest, mice were anaesthetised with pentobarbital sodium (50 mg/kg, intraperitoneal injection) until loss of consciousness was confirmed, followed by cervical dislocation to ensure death. Lungs were excised and lobes were separated for histopathology and lung homogenate preparation.

### Immunofluorescence staining of frozen sections

Frozen lung tissue sections were fixed in 4% paraformaldehyde for 15 min, washed with PBS, treated with 0.25% Triton X-100 for 10 min, and then blocked with 10% goat serum in 1% BSA for 30 min. Primary antibodies anti-CAP1 (1:100; H00010487-M02; Abnova) and anti-SPC (1:200; ab211326; Abcam) were incubated overnight at 4 ℃. After washing, sections were treated with secondary antibodies diluted in 1% BSA for 1 h in the dark. Finally, they were mounted with fluorescent medium and DAPI, and images were captured using a fluorescence microscope.

### Histology and immunohistochemistry

Lungs were fixed with 4% paraformaldehyde, paraffin embedded, sectioned, dewaxed in xylene, rehydrated in graded ethanol, and hematoxylin-eosin (H&E) stained using a standard procedure. Morphological changes were graded blindly by experienced histopathologists using the Ashcroft score (AS) scale [25]. For Masson’s trichrome staining the collagen was fixed in 4% paraformaldehyde, dehydrated through an ethanol series, paraffin embedded, sectioned and then stained. Immunohistochemistry was also performed on the fixed, paraffin embedded sections with anti-SPC (ab211326; Abcam) and anti-C5(sc-398247; Santa Cruz). Images were captured using a Konfoong scanner (KFBIO, Zhejiang, China).

### RNA library preparation and sequencing

Total RNA was extracted from the lung tissue using TRIzol^®^ Reagent, and quality was assessed with a 5300 Bioanalyser and quantified using the ND-2000. Only high-quality RNA (OD260/280 = 1.8–2.2, RIN ≥ 6.5, > 1 µg) was used for library construction at Shanghai Majorbio Bio-pharm Biotechnology Co., Ltd. The RNA-seq library was prepared using Illumina^®^ Stranded mRNA Prep with 1 µg of total RNA. mRNA was isolated via polyA selection and fragmented. Double-stranded cDNA was synthesized, followed by end-repair, phosphorylation, and ‘A’ base addition. Libraries were size-selected for 300 bp fragments and PCR amplified for 15 cycles. After quantification, paired-end sequencing was performed using the NovaSeq Xplus sequencer.

### Alignment and data analysis

Raw paired-end reads were trimmed and quality controlled using fastp [26]. Clean reads were aligned to the reference genome with HISAT2, and mapped reads were assembled with StringTie [27]. Differentially expressed genes (DEGs) were identified using the TPM method and quantified with RSEM [28]. Analysis was conducted using DESeq2 or DEGseq, considering DEGs with |log2FC| ≧ 1 and FDR ≤ 0.05 (DESeq2) or FDR ≤ 0.001 (DEGseq) as significant [29, 30]. Functional enrichment analyses for GO and KEGG were performed with Bonferroni-corrected P-value ≤ 0.05, using Goatools and KOBAS [31].

### Quantitative real-time PCR (qPCR)

Total RNA of tissues was isolated using Eastep ^®^ Super Total RNA Extraction Kit(catalog LS1040). After RNA extraction, 1000 ng of RNA was used to synthesize cDNA by Vazyme HiScript III RT SuperMix for qPCR (+ gDNA wiper) (catalog R323-01). Primers were synthesized by BGI (Beijing, China) and the sequence is listed in the Supplemental Table 1. Vazyme ChamQ Universal SYBR qPCR Master Mix (catalog Q711-02) was purchased and quantitative PCR was performed according to the manufacturer’s instructions. Relative levels of mRNA expression were calculated according to the 2^–ΔΔCT^ method. Individual expression values were normalized by comparison with GAPDH mRNA.

### Isolation of murine AT2

The murine lung tissue was digested into a single-cell suspension using collagenase IV (Roche catalog # 11088858001), and DNase I (Roche catalog # 10104159001). To isolate AT2 cells from mice, single-cell suspensions of murine lung tissue were stained and gated based on the following criteria: positive for EpCAM-APC (BioLegend catalog #118213) and negative for CD31-PE (BD Pharmingen catalog # 561073), CD45-PE (BD Pharmingen catalog # 561087), podoplanin-PE (Thermo catalog # 12-5381-80), Sca1-PE (BD Pharmingen catalog # 561076), CD24-PE (BioLegend catalog # 138503), and Zombie Green (BioLegend catalog # 423111).

### Micro RNA extraction

RNA was extracted from isolated AT2 cells using Tiangen RNAprep Pure Micro Kit, and whole RNA was used to synthesize cDNA. The subsequent procedure was the same as qPCR.

### Smart seq Library preparation and sequencing

Smart-seq of 300–1000 sorted AT2 cells from the mouse lungs was performed using the Clontech SMART-Seq v4 Ultra Low Input RNA Kit at Shanghai Majorbio. RNA-seq libraries were prepared with 10 ng total RNA. Reverse transcription was conducted using Oligo(dT) primers, followed by cDNA synthesis with TSO primers. The cDNA was amplified via PCR and tagged with linkers using Tn5 transposase. After quantification with Qubit 4.0, paired-end sequencing was performed on the NovaSeq X plus sequencer (2 × 150 bp read length). Data analysis was performed as previously described.

### CAP1 knockdown in A549 cells

Human lung cancer cell line A549 were purchased from the FDCC (Shanghai, China). A549 cells were grown in RPMI 1640 medium (Thermo, Suzhou, China) supplemented with 10% FBS (fetal bovine serum)(Gemini, USA) and 1% penicillin/streptomycin (Gemini, USA). A549 cells were transfected with CAP1-specific siRNA or negative control siRNA(Genomeditech, China) using Lipo 3000(Thermo Fisher Scientific) according to the manufacturer’s instructions. Cells were collected 48 h after transfection for subsequent experiments.

### JC-1 staining

Mitochondrial membrane potential was assessed using a JC-1 staining kit (Yeason, 40706ES60) according to the manufacturer’s instructions. Briefly, transfected A549 cells were incubated with JC-1 working solution at 37 °C for 20 min in the dark, followed by washing with buffer solution. Fluorescence images were captured using a fluorescence microscope, and the red/green fluorescence intensity ratio was quantified for analysis.

### ATP measurement

Intracellular ATP levels were measured using an ATP assay kit (Beyotime, S0026) following the manufacturer’s protocol. ATP levels were normalized to total protein concentration determined by BCA assay.

### Statistical analysis

The experimental groups and control group were compared by t-test or Mann-Whitney U test. Results are presented as mean±standard error or median(IQR). Survival curves were generated using the Kaplan–Meier method and compared using the log-rank (Mantel–Cox) test. *P*<0.05 was considered statistically significant. Graphpad Prism 9.0 (GraphPad, La Jolla, CA, USA) was used for statistical analyses.

## Conclusions

In summary, our study suggests that AT2 cell-specific CAP1 loss is associated with spontaneous pulmonary fibrotic remodeling, mitochondrial-related alterations, and altered local C5 signaling. These findings support a potential role of CAP1 in alveolar epithelial homeostasis and fibrotic lung disease and may provide insights into mechanisms relevant to pulmonary fibrosis progression.

## Supplementary Information

Supplementary Material 1.

## Data Availability

The RNA sequencing data generated in this study have been deposited in the NCBI Sequence Read Archive (SRA) under the project accession number PRJNA1165203, and can be accessed at: https://www.ncbi.nlm.nih.gov/bioproject/PRJNA1165203. Other datasets generated and/or analysed during the current study are included in this published article and its supplementary information files or are available from the corresponding author upon reasonable request.

## Abbreviations

CAP1: Cyclase-associated protein1
AT2: Alveolar type 2 cell
C5: Complement 5
CKO: Conditionally knock out
FVC: Forced Vital Capacity
FEV_0.05_: Forced Expiratory Volume at 0.05 s
