# Supplementary material for: Type II alveolar epithelial cell-specific CAP1 loss is associated with spontaneous pulmonary fibrosis and altered complement 5 signaling
Source: BMC Pulm Med. 2026 Jun 9;26:359. doi: 10.1186/s12890-026-04384-y (PMC13483859; doi:10.1186/s12890-026-04384-y)
Supplement: Supplementary file 1 [file 12890_2026_4384_MOESM1_ESM.doc]

### Supplemental material

Specific primers for genotyping

The following primers were used for CAP1 flox genotyping: (F1: 5’-TCCTGTCCTTTCTTTAGCAGAATGA-3’, R1: 5’-TCTCTGGGCTTAATGTTCTTCATC-3’). For homozygous was one band with 267 bp, for heterozygous was two bands with 267 bp and 198 bp, for wt was one band with 198 bp. To detect the presence of Sftpc-CreRT2, the following two primers were used: cre-F (5’-CAGGTGAGCAACAGTACCTTTCAG-3’) and cre-R (5’-CTAGGAATGCTCGTCAAGAAGACAG-3’). Mutant: 494 bp. The tissue-specific gene deletion can be confirmed by the following primers: (F1: 5’-TCCTGTCCTTTCTTTAGCAGAATGA-3’, R2: 5’-TCCAGAAGAGATGCAGAGTGTCAG-3’). If with Cre activity, there was one band with 315 bp.

### Supplemental table 1

qPCR primers

| Gene | Primers |
| --- | --- |
| m-Col1a1 | GACAGGCGAACAAGGTGACAGAG |
|  | CAGGAGAACCAGGAGAACCAGGAG |
| m-Col1a2 | GGCAACAGCAGGTTCACCTACTC |
|  | GTCAGCACCACCAATGTCCAGAG |
| m-Fn1 | AGTGGCTGAAGTCGCAAGGAAAC |
|  | TAAGTCTGGGTCACGGCTGTCTC |
| surfactant protein C | TCCTCGTTGTCGTGGTGATTGTAG |
|  | ATGCTCATCTCAAGGACCATCTCAG |
| m hemolytic complement | GACCTTCACCCTGCTTCCTCTG |
|  | CTCTTGACTCCTTCTGGCACTACC |
| m component 6 | TTTGATGACTTCGGGACCCACTAC |
|  | TGGCGGCTGAATTGGTAGATAAGG |
| m component 7 | CAGCACTCCACCACCAGCAG |
|  | TCATCTCGGGCACATACATCCAAG |

### Supplemental table 2

Expression of genes in AT2 cells of CAP1 CKO mice and controls after 2 weeks of tamoxifen injection

| GENES | CKO AT2(n=2) | Control AT2(n=1) | log2FC | Pvalue | Padjust | Significant | Regulate |
| --- | --- | --- | --- | --- | --- | --- | --- |
| CAP1 | 0 | 1.15 | -7.320796236 | 0.037559632 | 1 | yes | down |
| Hc | 0 | 68.03 | -14.23489337 | 0.003998728 | 1 | yes | down |
